# Comprehensive annotation of the enzymes of *Drosophila melanogaster*

**DOI:** 10.1093/g3journal/jkaf294

**Published:** 2025-12-08

**Authors:** Phani V Garapati, Rossana Zaru, Helen Attrill, Gilberto dos Santos, Josh Goodman, Jim Thurmond, Steven J Marygold

**Affiliations:** FlyBase, Department of Physiology, Development and Neuroscience, University of Cambridge, Cambridge, Cambridgeshire CB2 3DY, United Kingdom; FlyBase, Department of Physiology, Development and Neuroscience, University of Cambridge, Cambridge, Cambridgeshire CB2 3DY, United Kingdom; FlyBase, Department of Physiology, Development and Neuroscience, University of Cambridge, Cambridge, Cambridgeshire CB2 3DY, United Kingdom; FlyBase, Department of Molecular and Cellular Biology, Harvard University, Cambridge, MA 02138, United States; FlyBase, Department of Biology, Indiana University, Bloomington, IN 47405, United States; FlyBase, Department of Biology, Indiana University, Bloomington, IN 47405, United States; FlyBase, Department of Physiology, Development and Neuroscience, University of Cambridge, Cambridge, Cambridgeshire CB2 3DY, United Kingdom

**Keywords:** *Drosophila*, enzymes, Gene Ontology, functional annotation, FlyBase, pseudoenzymes, paralogs, FlyCyc, metabolism

## Abstract

We have completed a systematic survey of *Drosophila melanogaster* enzymes, improving the coverage and accuracy of their functional Gene Ontology annotations in FlyBase and collaborating databases. We made >5,000 changes to manual Gene Ontology annotations by reviewing information from the literature and consulting expert databases, resulting in the final verification of 3,708 *Drosophila* enzyme-encoding genes. Herein, we present an overview of the enzyme landscape in *Drosophila*, including insights on enzyme paralogs, pseudoenzymes, and enzymatic complexes, and compare these with corresponding datasets for yeast and humans. We also show how the presentation of enzyme data on FlyBase gene reports has been enhanced, including the addition of Enzyme Commission (EC) information and RHEA reaction graphics. For each class of enzyme, we have created a “Gene Group” page in FlyBase to tabulate the group members and facilitate access to related information and tools. Together, this work provides a comprehensive enzyme resource to serve the *Drosophila* research community and beyond. As a practical example of its utility, we used our improved dataset to update the FlyCyc model of *Drosophila* metabolism.

## Introduction

A complete and accurate record of the complement of enzymes encoded by a genome is critical for understanding the biology of the organism. It enables metabolic network construction, explains physiological differences, informs evolutionary analyses, and has many biomedical and industrial applications. As such, enzymatic data are accessed and utilized extensively by researchers, bioinformaticians, industry, and increasingly by AI-based tools. However, an incomplete or inaccurate annotation of the catalytic potential of a genome can result in experimental errors, misleading conclusions, and missed opportunities.

Enzymes and the reactions they catalyze are denoted in biological databases using several different methods. First, the Enzyme Commission (EC) system uses a hierarchical, 4-digit numbering system to classify enzymes based on their reaction mechanism (McDonald and Tipton 2023). For example, EC:2.-.-.- describes “transferases”, EC:2.7.-.- is “phosphotransferases”, EC:2.7.1.- is “phosphotransferases with an alcohol group as acceptor”, and EC:2.7.1.11 is the specific enzyme “6-phosphofructokinase”. The EC system is fundamental to metabolic databases like KEGG (Kanehisa et al. 2025) and MetaCyc (Karp et al. 2019) and is also used to directly annotate proteins in UniProtKB (Bansal et al. 2022). Second, genes/gene products may also be annotated with the specific reaction(s) they catalyze using a reaction identifier in KEGG, MetaCyc, or RHEA (Bansal et al. 2022). RHEA IDs are used alongside EC numbers to annotate enzymes in UniProtKB, whereas KEGG and MetaCyc reaction IDs are used within their respective databases. Third, enzymes may be annotated with catalytic activity terms from the molecular function aspect of the Gene Ontology (GO), the organization of which largely follows the EC hierarchy (Gene Ontology Consortium 2023). The GO is widely used to annotate gene product functions across biological knowledge bases, including UniProtKB and model organism databases. With all these methods, enzymatic annotations may be based on direct experimental evidence or inferred indirectly from a source annotation made to an ortholog. This latter approach is a powerful way to transfer knowledge between species and fill annotation gaps, though appropriate caution needs to be taken when interpreting such annotations.

Despite the importance and widespread use of these datasets, studies comparing enzyme annotations in different databases or using different methods have reported significant discrepancies (Schnoes et al. 2009; Griesemer et al. 2018 ; Garapati et al. 2019). Some of these discrepancies are explained by variations in database update frequency or the sources/strategies of annotation used, but they are largely due to the presence of erroneous annotations or the omission of correct annotations. Left unchecked, such false positive and false negative annotations cause significant downstream issues for enzyme-related research and data analysis.

With this in mind, we have undertaken a systematic review of all the enzyme-encoding genes of the model organism *Drosophila melanogaster*. *Drosophila* has a long history of enzyme research, from early genetic studies of eye and body color mutants and biochemical purifications of enzymatic activities, to more recent molecular-genetic investigations of kinases and phosphatases that regulate key signaling pathways (Garapati et al. 2019). Moreover, the recent surge in the use of *Drosophila* to study metabolism (Supplementary Fig. 1) underscores the need for accurate enzyme annotation in this organism. We have reviewed almost 4,500 *Drosophila* genes described as having catalytic activity based on existing annotations and/or their orthology to well-characterized enzymes in humans or yeast: ∼650 genes were found to be misannotated as enzymes, and ∼450 genes were missing enzymatic annotations. Absent GO annotations have since been added, and all problematic annotations have been fixed at source. We conclude that the *Drosophila* genome encodes 3,708 enzymes, a third of which are currently supported by experimental evidence. Furthermore, we have used the improved GO annotations to update the FlyCyc metabolic model and have also added several new features to the FlyBase website to enhance access to enzymatic data.

## Methods

### Enzyme annotation review

**Fig. 1.**
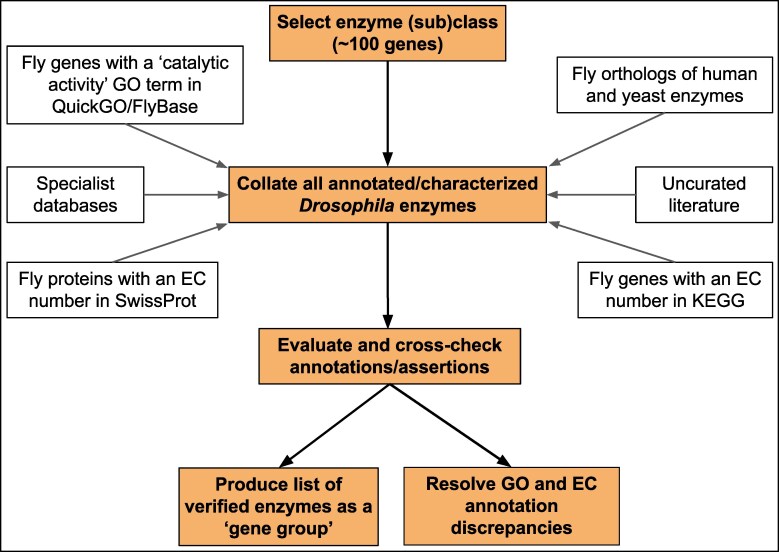
Overview of the strategy used to review *Drosophila* enzyme annotations.

Enzymatic annotations to *Drosophila* genes were obtained, analyzed, and assessed as described (Garapati et al. 2019) and summarized in Fig. 1. The majority of enzymatic terms in the GO are grouped under “catalytic activity” (GO:0003824), though translocase terms are children of “primary active transmembrane transporter activity” (GO:0015399). Custom scripts were written to automate cross-checks between catalytic GO annotations and enzymatic Gene Groups with each FlyBase release and generate reports for manual inspection. Errors or omissions in the GO were reported on the go-ontology GitHub repo (https://github.com/geneontology/go-ontology) and addressed by GO editors. Errors or omissions in GO annotations were either fixed directly or reported on the go-annotation repo (https://github.com/geneontology/go-annotation) for other curators to resolve. Data pertaining to *Drosophila* gene nomenclature, GO annotations, enzymatic complexes, paralogs, and testis-specific expression were obtained from FlyBase (FB2025_04) using a combination of ID Validator, QuickSearch, HitList, and Batch Download tools (Öztürk-Çolak et al. 2024). Pseudoenzymes were identified based on GO annotations, published data, or manual sequence inspection using the UniProt Align tool (https://www.uniprot.org/align) as described (Zaru and Marygold 2025). Basic list comparisons were performed using tools developed by the Bioinformatics and Research Computing group at the Whitehead Institute (http://barc.wi.mit.edu/tools/).

### Cross-species comparisons

The AmiGO 2 web server (https://amigo.geneontology.org/amigo/) was queried (2025 August 25) for the high-level catalytic GO terms shown in Table 1 to generate a table of annotations using each GO term or any of its children. The resulting annotation table was filtered for the desired “Organism” (*Homo sapiens*, *Saccharomyces cerevisiae*, or *Drosophila melanogaster*) and “Type” (proteins only); negative annotations (i.e. those using an “Annotation qualifier” of “not”) were excluded. Annotations were then downloaded and the contents of the bioentity ID column processed to obtain counts of unique genes/proteins. The same method was used to calculate counts of enzymes annotated to be part of complexes, except that “Type” was changed to “protein_complex.” Total counts of protein-coding genes in each organism were obtained by using the main search tool on the homepage of the Alliance of Genome Resources (https://www.alliancegenome.org/) version 8.1.0 (Alliance of Genome Resources Consortium 2024). Initially, all genes were found by selecting the “Gene” category and performing a blank search. This was followed by filtering for the “Biotype” of “protein coding gene” and the desired “Species.”

**Table 1.** Sequence-localized Drosophila genes annotated to the major enzyme classes in FlyBase.

Enzyme class (GO id/EC number)	Number of genes annotated with catalytic GO terms			Number of genes in ENZYMES gene group (FB2025_04)
	Before (FB2017_05)	Now (FB2025_04)	+/−	
Oxidoreductases (GO:0016491/EC:1)	612	578	78/112	554
Transferases (GO:0016740/EC:2)	1,316	1,266	194/244	1,250
Hydrolases (GO:0016787/EC:3)	1,765	1,513^a^	211/463	1,409
Lyases (GO:0016829/EC:4)	117	133	30/14	131
Isomerases (GO:0016853/EC:5)	96	256^a^	169/9	250
Ligases (GO:0016874/EC:6)	111	149	54/16	128
Translocases (GO:0015399/EC:7)	133	133	45/45	107
**Total (unique)** ^b^	**4,015**	**3,810**	**452**/**657**	**3,708**

The first 4 columns show the number of Drosophila genes with GO annotations to the relevant activities before (FlyBase release FB2017_05) and after (FB2025_04) the review, specifying the number of annotated genes that were added (+) or removed (−) in each class. The final column shows the final number of manually validated enzyme-encoding genes, as reported within the ENZYMES gene group in FlyBase (FB2025_04). The bold values merely distinguish the ‘Total’ line from all other lines.

^a^The large changes in hydrolase and isomerase annotations were largely due to reclassification of motor/helicase activities within the GO to align with EC classification.

^b^The “Total (unique)” number is not the sum of the preceding rows because some enzymes classify under multiple activities, and some catalytic GO terms are not classified under the 7 major groupings.

### FlyCyc update

FlyCyc was generated using the PathoLogic component of the Pathway Tools software (Karp et al. 2011) and the validated GO annotations to *Drosophila* enzymes contained in FlyBase. The process will be described in more detail elsewhere.

### GitHub issue analysis

Enzyme-related issues made during this project were retrieved by searching the go-ontology repository (https://github.com/geneontology/go-ontology/issues) with the filter set “is:issue label:enzymes created:>2017-01-01 created:<2025-10-02 (author:sjm41 OR author:phanivg OR author:hattrill OR author:rozaru)” or the go-annotation repository (https://github.com/geneontology/go-annotation/issues) with the filter set “is:issue created:>2017-01-01 created:<2025-10-02 (author:sjm41 OR author:phanivg)”. Further classification was achieved by adding additional label filters (e.g. label:“term name” or label:“InterPro mapping”) as appropriate, and counts of closed/open issues were recorded.

### Counts of papers about Drosophila metabolism

The QuickSearch tool on FlyBase (Marygold and FlyBase Consortium 2023) was used to calculate counts and proportions of research papers included in the FlyBase bibliography that mention “metabolism” or related words in their title and/or abstract. Specifically, the “References” tab of QuickSearch was selected with “Publication type” set to “paper”, the “Year” set to “2000 to 2024” and (optionally) the “Title/Abstract” field set to “metaboli*”. From the resulting HitList, the “Analyze” feature was used to analyze the frequency of values by year.

## Results and discussion

### Systematic enzyme review and annotation

Our general approach for identifying, reviewing, and, where necessary, re-annotating *Drosophila* enzyme-encoding genes was published previously (Garapati et al. 2019) and is summarized in Fig. 1. Briefly, we consulted and compared existing GO and EC annotations from multiple sources for each enzyme class/subclass. Discrepancies between sources were carefully examined—erroneous GO annotations were corrected/deleted, or missing GO annotations added, as appropriate. Experimental evidence from uncurated literature was sought to support the annotated activity wherever possible. High-quality enzyme annotations to human and yeast were also obtained and mapped to *Drosophila* orthologs to infer GO annotations that were absent by other methods. Overall, this exercise added >4,000 new manual GO annotations, removed >1,000 incorrect or redundant annotations, and updated >900 existing annotations.

During this project, >700 tickets were submitted to the GO GitHub tracker to correct or improve aspects of the ontology, and >500 tickets were made in the GO annotation GitHub tracker to fix computational annotation pipelines (Supplementary Fig. 2). Over 99% of these tickets have been addressed. This holistic approach had 2 benefits beyond fixing the immediate issue with a *Drosophila* annotation: it minimized the likelihood that the same annotation problem would reoccur in future, and the underlying issue was corrected for all affected annotations in all species. For example, half of the GO annotation tickets reported issues with the “InterPro2GO” pipeline, which associates GO terms with InterPro signatures and thereby adds GO annotations across many species. While this pipeline is very useful for propagating knowledge and filling annotation gaps, any errors in the source annotation or ignorance of taxa-specific differences generate many false positive annotations.

In this way, a set of verified GO annotations was compiled for each of the major enzyme classes. These include the oxidoreductases, transferases, hydrolases, lyases, isomerases, and ligases (that correspond to EC classes 1 to 6, respectively), as well as the translocases (EC:7) that catalyze transmembrane transport reactions typically coupled to ATP hydrolysis (e.g. ABC-type transporters) or oxidoreduction reactions (e.g. respiratory chain complexes). Table 1 summarizes the number of *Drosophila* genes annotated to each class before and after the review, and the number of genes that were removed or added to each class. Overall, the total number of genes annotated to catalytic GO terms did not change substantially, reducing by ∼200 genes. However, that metric hides the reality that 657 genes had erroneous catalytic GO annotations removed, and 452 genes were newly annotated to catalytic GO terms. False positives were mainly due to errors in computational annotation pipelines and/or annotations to noncatalytic subunits of enzymatic complexes, whereas false negatives were mostly the result of missing relations/cross-references in the ontology or uncurated literature (Garapati et al. 2019). The verified sets of GO annotations are now included in the FlyBase database as well as other databases that consume our annotations (e.g. Alliance of Genome Resources, AmiGO, QuickGO, UniProtKB).

Although GO annotation is the primary method to describe gene product function in FlyBase and most other biological databases (Gene Ontology Consortium 2023), it does not allow for categorization of *Drosophila* enzymes at the level of stability or detail we wished to achieve. For example, requested additions/edits to the GO or GO annotations can take several FlyBase releases to implement; inappropriate GO annotations derived from computational pipelines (e.g. InterPro2GO) may be difficult to challenge; GO annotations based solely on high-throughput experiments may be unreliable; and certain useful enzyme classifications are outside the scope of the GO. For these reasons, we decided to maintain independent lists of our manually verified enzymes using the existing “Gene Group” infrastructure within FlyBase (Attrill et al. 2016). FlyBase gene groups are manually curated lists of functionally related *Drosophila* genes arranged in a hierarchical fashion, accompanied by a hand-written summary and explanatory comments, and associated with relevant literature. We created >750 enzymatic gene groups in FlyBase, representing groupings from the top-level ENZYMES group, through high-level subgroups such as HYDROLASES and LIGASES, down to terminal groups that correspond to specific GO/EC terms such as CHITINASES or DNA LIGASES. As shown in Table 1, the number of genes verified to belong to enzymatic gene groups differs from that annotated with catalytic GO terms—this is due to reasons described above, mainly the presence of false positive GO annotations from computational pipelines. Importantly, the gene group framework accommodates groupings that are too specific for GO classification but are nonetheless useful for categorization and presentation to users. For example, the 451 *Drosophila* genes encoding peptidases have been organized into gene groups based on their family membership in the MEROPS database (Rawlings et al. 2018); the 219 genes encoding protein kinases have been classified into gene groups based on the families in KinBase (Manning et al. 2002); the ∼90 genes coding for cytochrome P450 enzymes belong to 4 different gene groups based on the classification by Feyereisen (Dermauw et al. 2020); and whereas the GO only has one main term for “GTPase activity,” the gene group structure partitions the 146 GTPase-encoding genes into 10 functional subgroups, including a further subdivision of the 80 members of the Ras GTPase superfamily (Wennerberg et al. 2005).

Many groups of *Drosophila* enzymes have received scant or no attention prior to our systematic review, and we have published several papers reporting in-depth analyses of such cases (Lu et al. 2015; Marygold et al. 2020a, 2020b; Ahn and Marygold 2021; Meyer et al. 2021; Marygold 2024; Zaru and Marygold 2025). The remainder of this report provides a more general overview of the entire enzyme landscape in *Drosophila*, highlighting improvements to underlying enzymatic annotations and showcasing enhancements to the FlyBase and FlyCyc websites.

### Overview of the *Drosophila* enzyme landscape

**Fig. 2.**
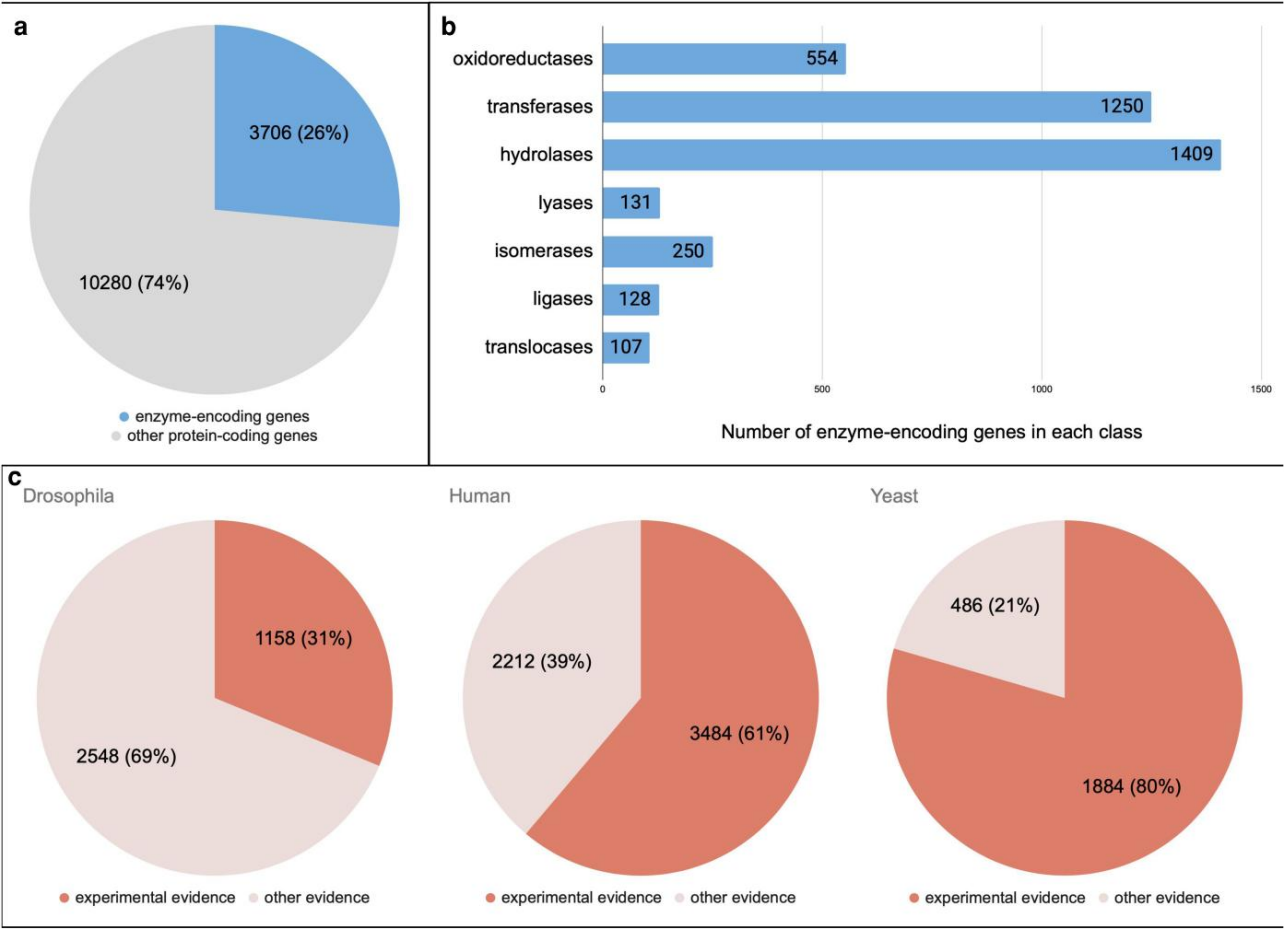
Summary statistics for the verified enzyme-encoding genes in the *Drosophila* genome. a) Proportion of protein-coding genes that encode enzymes in the *Drosophila* genome. b) Distribution of *Drosophila* enzyme-encoding genes across the 7 major classes. c) Proportion of *Drosophila* enzyme-encoding genes for which the catalytic activity is based on experimental evidence, compared to that of human and yeast.

The completion of our enzyme annotation review enables, for the first time, an accurate overview of the catalytic capabilities encoded by the *Drosophila* genome (Fig. 2). There are 3,708 validated enzyme-encoding genes within the ENZYMES gene group, which includes 2 RNA genes encoding ribozymes (*RNaseP:RNA* and *RNaseMRP:RNA*), meaning that 26% of all protein-coding genes encode enzymes. This figure is similar to that of other well-annotated eukaryotic genomes: for example, 29% of the human genome and 36% of the yeast (*S. cerevisiae*) protein-coding genome encode enzymes. Hydrolases constitute the largest class of *Drosophila* enzymes (38%), followed by transferases (34%) and oxidoreductases (15%), with the other enzyme classes having smaller representation. Again, this division is largely mirrored in yeast and humans, suggesting it is representative among eukaryotes. Notably, a relatively small proportion (31%) of *Drosophila* enzymes have direct experimental evidence for their annotated catalytic activity—most activities are inferred from proven activities in homologous enzymes in other species,that is, via sequence similarity, shared evolutionary ancestry, or the presence of protein domains. A much greater proportion of enzymes in humans and yeast have experimental evidence for their given activity (61% and 80%, respectively). Indeed, yeast and human orthologs were frequently used as the source for inferring an enzymatic activity of fly gene products in our annotation review.

### Special features of the *Drosophila* enzyme set

Although the enzyme landscape of *Drosophila* is similar to that of yeast and humans at a gross level, important differences become evident when examined at finer detail. For example, a comparison of annotations to specific (“leaf”) catalytic GO terms (that often correspond to 4-digit EC numbers) reveals that 64 enzymatic activities are present in *Drosophila* but absent in yeast and humans, suggesting they act in insect or arthropod-specific processes (Supplementary Table 1, tab 1). Indeed, many of these genes are involved in pathways that have no physiological equivalent in yeast or humans, such as cuticle melanization (e.g. *tan*, *ebony*, *yellow-f*), metamorphosis/molting (e.g. *Juvenile hormone esterase*, *shade*, *pinkman*) or compound eye pigmentation (e.g. *white*, *sepia*, *Dihydropterin deaminase*). It is also evident that *Drosophila* have a relatively higher complement of “oxidoreductases acting on paired donors, with incorporation or reduction of molecular oxygen” (EC:1.14.-.-) (Supplementary Table 1, tab 3). This is largely explained by flies having an exceptionally large number of genes encoding peptidyl-proline dioxygenases (PPDOXs) and cytochrome P450 enzymes (Abrams and Andrew 2002; Dermauw et al. 2020). PPDOXs hydroxylate proline in collagens within the extracellular matrix (ECM). Whereas humans generate diversity in their ECM via tissue-specific expression of an expanded number of collagen genes, insects appear to achieve this via tissue-specific expression of 28 different PPDOX genes. P450 enzymes have roles in many physiological processes but are particularly associated with the detoxification of xenobiotics including insecticides, which likely explains their prevalence in flies.

A similar analysis highlights 57 enzymatic activities that are conserved in both yeast and humans but are conspicuously absent in *Drosophila* (Supplementary Table 1, tab 2). For example, telomerase and other telomere-maintenance enzymes are absent in *Drosophila* since insects maintain their telomeres using a different, transposable element-based system (Pardue and DeBaryshe 2003). The fact that several conserved enzymes catalyzing sterol biosynthesis are not found in *Drosophila* is consistent with their being sterol auxotrophs, meaning they must obtain sterols from the diet (Carvalho et al. 2010). *Drosophila* also lacks several of the enzymes involved in the metabolic pathway synthesizing NAD+ *de novo* from tryptophan—it appears that insects have lost this pathway and instead rely exclusively on synthesizing NAD+ via salvage pathways (Gossmann et al. 2012). Glutathione reductase is also absent in flies—its function in antioxidant defense has been replaced by a thioredoxin-based system (Kanzok et al. 2001).

### Enzyme paralogs

Gene duplications within a genome, resulting in paralogs, are common in eukaryotes (Reams and Roth 2015). We used the implementation of paralog data in FlyBase, which is derived from the DIOPT resource (Hu et al. 2022), to examine the prevalence of paralogs of enzyme-encoding genes in *Drosophila*. DIOPT integrates *Drosophila* paralog predictions from 13 different resources and assigns a score to each paralog pair corresponding to the number of resources supporting that call. Using a relatively stringent cut-off score of 7, there are 3,866 (28%) genes in the entire *Drosophila* protein-coding genome that have one or more paralogs (Supplementary Table 2, tab 1). Strikingly, almost half of those genes (1,880) are included within our list of manually verified enzyme-encoding genes (Supplementary Table 2, tab 2). Viewed another way, 51% of enzyme-encoding genes have at least one paralog. Thus, the existence of paralogous enzyme-encoding genes is common, occurring at around twice the frequency seen for all protein-coding genes.

Most of the paralogous groups of enzyme-encoding genes contain between 2 and 4 genes, but some large gene families (e.g. cytochrome P450, UDP-glycosyltransferases, glutathione S-transferases, serine proteases, ecdysteroid kinase-like enzymes) include 20 or more paralogs (Supplementary Table 2, tab 3). Notably, one or more paralogs within a group may exhibit tissue-specific (often testis-specific) expression (Supplementary Table 2, tab 2; Zaru and Marygold 2025) and/or may be catalytically inactive, as described below. This suggests that some paralogs have diverged to have tissue-specific or modulatory functions. Interestingly, other paralogous genes have diverged sufficiently to adopt different catalytic activities. For example, *Alas* and *Gcat* are paralogs that encode acyltransferases (EC:2.3.1.-), but whereas Alas is an aminolevulinate synthase (EC:2.3.1.37), Gcat has glycine C-acetyltransferase activity (EC:2.3.1.29).

### Pseudoenzymes

Pseudoenzymes are similar in amino acid sequence to functional enzymes but lack key residues in their catalytic, substrate-binding, and/or cofactor-binding site(s) that render them catalytically inactive (Goldberg and Sreelatha 2023). Many pseudoenzymes are remarkably well conserved and have evolved essential catalytic-independent functions. For example, Tribbles is a conserved pseudokinase that has evolved a scaffolding/adaptor activity, whereas Rpn8 is a conserved pseudopeptidase that regulates Rpn11 deubiquitinase activity within the proteasome (Goldberg and Sreelatha 2023). In *Drosophila*, several pseudoenzymes have been characterized through direct experimentation, and more have been inferred by virtue of their lack of conserved sequence features. However, our enzyme review revealed that many *Drosophila* pseudoenzymes were either wrongly annotated with a GO enzymatic activity term or otherwise difficult to identify in FlyBase and other databases.

The conventional method to record an observed/predicted absence of catalytic activity within biological databases like FlyBase is to annotate with the appropriate GO term in conjunction with a “NOT” qualifier, for example “NOT peptidase activity.” Prior to our annotation review, there were 151 *Drosophila* genes with a NOT annotation to a term within the “catalytic activity” branch of the GO (Table 2). Following our review, 21 of these genes had their NOT annotation removed, either because the original assertion/annotation was erroneous or a subsequent study demonstrated the presence of catalytic activity. More significantly, an additional 218 genes were newly annotated with a “NOT catalytic activity” annotation, most of which are pseudopeptidases (also known as “nonpeptidase homologs”), based on previously uncurated information in the literature or the MEROPS peptidase database (Rawlings et al. 2018).

**Table 2.** Pseudoenzyme annotations in FlyBase.

Pseudoenzyme class (GO id)	Number of “NOT” GO-annotated genes			Number in PSEUDOENZYMES gene group (FB2025_04)
	Before (FB2017_05)	Now (FB2025_04)	+/−	
Pseudopeptidases (NOT GO:0008233)	61	184	134/10	180
Pseudokinases (NOT GO:0016301)	30	43	16/3	40
Pseudophosphatases (NOT GO:0016791)	11	10	0/1	8
Other pseudoenzymes	49	111	68/7	85
**Total**	**151**	**348**	**218**/**21**	**313**

The first 4 columns show the number of *Drosophila* genes with negative annotations (i.e. qualified with “NOT”) to catalytic activity GO terms before (FB2017_05) and after (FB2025_04) the review, specifying the number of annotated genes added (+) or removed (−) in each class. The final column shows the final number of manually validated pseudoenzyme-encoding genes, as reported within the PSEUDOENZYMES gene group in FlyBase (FB2025_04). The bold values merely distinguish the ‘Total’ line from all other lines.

However, the presence of a “NOT catalytic activity” annotation alone is not diagnostic of a pseudoenzyme. This is because the convention may also be used to record that an otherwise active enzyme doesn’t work with a particular substrate or doesn’t show a certain specific activity. For example, the *Drosophila Argp* gene is annotated with “NOT protein tyrosine phosphatase activity” to indicate that the encoded protein lacks this activity despite the presence of protein tyrosine phosphatase domains, but it also has a positive “protein arginine phosphatase activity” annotation because the protein has been experimentally shown to have that related activity. Additional drawbacks of the “NOT” annotation convention is that researchers may not be aware of it, and that different websites choose to display/filter NOT annotations in different ways. Thus, we decided to assemble a PSEUDOENZYMES gene group within FlyBase, distinct from the ENZYMES gene group, to clearly distinguish these genes (Table 2). The PSEUDOENZYMES group contains a total of 313 genes that encode proteins with at least one inactive catalytic domain, with the majority classified as pseudopeptidases, pseudokinases, or pseudophosphatases. Currently, 8% of all genes encoding enzyme-like proteins in *Drosophila* are characterized as pseudoenzymes, which is in line with findings in other species (Ribeiro et al. 2019). However, we expect this proportion to grow in future as additional *Drosophila* enzyme paralogs are analyzed in greater detail.

### Enzymatic complexes

Many enzymes operate within heteromeric macromolecular complexes, wherein they interact with noncatalytic subunits that serve regulatory or scaffold functions. Gene products that act within such complexes are all annotated with the GO cellular component term “catalytic complex” (GO:1902494) or a child thereof, e.g. “pyruvate dehydrogenase complex” (GO:0045254). By cross-referencing our enzyme list with “catalytic complex” annotations in FlyBase, we find that 422 (11%) enzymes operate within a protein complex (Supplementary Table 3). This is likely to be an underestimate as we have not yet systematically reviewed/annotated all such catalytic complexes in *Drosophila*. Such annotation efforts have been undertaken for the human and yeast genomes, where 16% and 24% of enzymes, respectively, are annotated to be part of a complex by the same criteria.

### Orphan enzyme-encoding genes

We encountered 71 “orphan enzyme genes” during our annotation review, defined as genes having a catalytic GO annotation and/or an enzyme-related name but not localized to the sequenced genome (Supplementary Table 4). Most such genes were originally created in FlyBase based on older, pregenomic research that isolated/assayed an enzymatic activity but did not identify the responsible gene/protein. Using our revised set of GO annotations, along with any available mapping data and subsequently published information, we were able to associate 37 of these orphan genes with sequenced-localized (“CG”) genes. This is very useful to researchers because it integrates all relevant biochemical, genetic, and publication data into a single FlyBase report. A smaller number (25) of orphan enzyme gene reports were deleted from FlyBase since they contained no useful information and/or were based solely on an unpublished account (e.g. a conference abstract).

### Gene nomenclature updates

Our annotation review also identified many enzyme-encoding genes in FlyBase that were previously unnamed; that is, they were known only by their “CG” number. We were able to name 458 of these using standardized enzyme nomenclature based on previously uncurated information in the literature and/or unambiguous orthology to human genes (Supplementary Table 5). In addition, another 139 enzyme-encoding genes with existing symbols/names were renamed to rationalize the nomenclature within a given enzyme class and/or reflect the preferred usage in the fly literature. Although catalytic functions of gene products are better described by their functional annotations, an accurate and informative symbol is still important and useful, especially when viewing gene lists or conducting cross-species analyses.

### Enzyme-related improvements to the FlyBase website

**Fig. 3.**
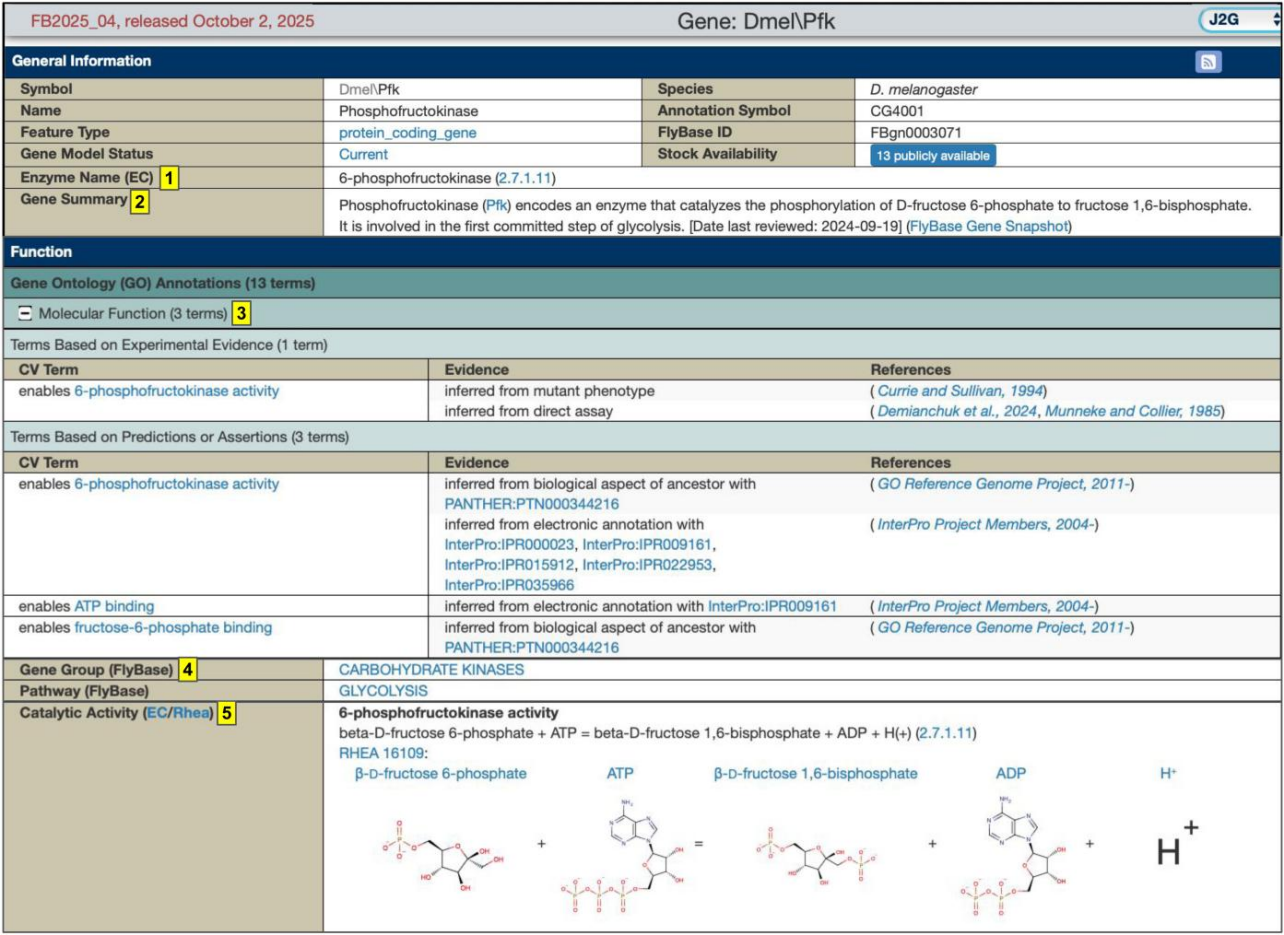
Enzyme-related enhancements to FlyBase gene reports, as shown here for the *Phosphofructokinase* (*Pfk*) gene. (i) The EC name and number are now shown. (ii) Hand-written “Gene Snapshots” have been improved. (iii) Catalytic GO annotations have been improved for accuracy and coverage, adding experimental evidence whenever available. (iv) Links to related enzyme Gene Group pages are included. (v) RHEA reaction IDs and corresponding graphics are shown.

The work described above has enabled several enhancements to the representation of enzymes in FlyBase. First and foremost, the improvements to GO annotations and gene nomenclature have been incorporated into individual gene reports (Fig. 3). Second, gene reports have been augmented with 2 new fields for enzyme-encoding genes: the EC name and number (where this corresponds to a complete, 4-digit EC number) and a graphic imported from the RHEA database illustrating the reaction catalyzed by the enzyme at the molecular level (Fig. 3). These EC and RHEA annotations are computed based on the EC/RHEA cross-references to the annotated GO molecular function term, thereby ensuring consistency between these 3 sources without any additional curation work. (Note that these cross-references are highly accurate, in part because of the >350 GO tickets made during this project to correct erroneous or add missing EC/RHEA cross-references [Supplementary Fig. 2].) Third, many “gene snapshots”, which are short, hand-written summaries of the main functions of *Drosophila* genes, have been written anew or updated for enzyme-encoding genes.

**Fig. 4.**
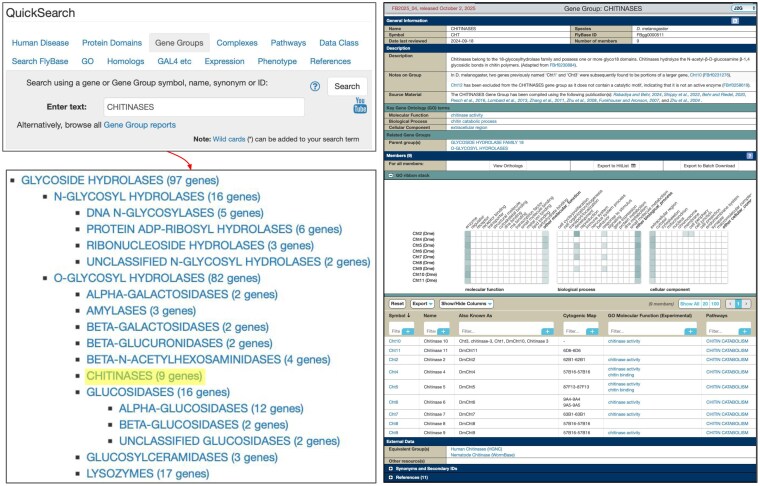
Gene Group reports for *Drosophila* enzymes. Left: These reports can be searched via the “Gene Groups” tab of the QuickSearch tool or browsed by clicking the browse link. Right: The CHITINASES Gene Group report is shown as an example.

A fourth improvement is the provision of comprehensive Gene Group reports for all *Drosophila* enzyme classes and subclasses (Fig. 4, right-hand panel). As mentioned, these reports are a general solution in FlyBase to maintaining stable lists of functionally related genes, arranged in a hierarchical fashion and associated with useful links and tools (Attrill et al. 2016). The central feature of these reports is an interactive table listing the members of the group, with options to view orthologs of the members or export the list to another FlyBase tool for further analysis. A GO ribbon stack sits above the table and gives an overview of functional annotations associated with the group, where the depth of color of a cell correlates with the number of annotations for the given GO class. Other useful features within these reports include a hand-written textual description of the group, a listing of literature used to compile the group, explanatory notes about the group such as paralogs or related pseudoenzymes, links to parent/child groups within FlyBase, and links to equivalent gene groups at other resources, notably human gene groups hosted by the HGNC (Seal et al. 2023). All enzymatic groups may be searched or browsed by selecting the “Gene Groups” tab of the main QuickSearch tool on the FlyBase homepage (Fig. 4, left-hand panels).

Finally, bulk access to enzymatic data has been improved by providing a new precomputed file of enzyme data (dmel_enzyme_data_fb_*.tsv) that lists all verified *Drosophila* enzymes together with their gene group, GO molecular function and EC annotations. This is available via the “Downloads” link on any FlyBase page and can also be used as a data source when using the FlyBase Batch Download tool.

### Use case: updating FlyCyc

Genome-scale metabolic models (GEMs) represent the network of all metabolic reactions and pathways present in a given species. They provide a powerful infrastructure to simulate the effects of perturbing the levels/activities of individual enzymes, reactions, or metabolites on the fitness of the organism (Gu et al. 2019). The quality, and therefore the ultimate utility, of a GEM depends critically on the quality and coverage of the component enzyme/reaction annotations. Although several GEMs for *Drosophila* exist, they all use orthology-based approaches and reconstruct *Drosophila* metabolism using generic templates from KEGG and/or MetaCyc (Büchel et al. 2013; Wang et al. 2021; Cesur et al. 2023). That is, these GEMs ignore the *Drosophila*-specific and manually curated/verified enzyme annotation data available in FlyBase. We therefore used our reviewed set of GO annotations for *Drosophila* enzymes to compute a high-quality, annotation-based *Drosophila* metabolic pathway database within the BioCyc infrastructure (Karp et al. 2019), suitable for building improved *Drosophila* GEMs in future.

This database, known as FlyCyc, had been computed once before in 2008 based on the FlyBase GO annotation set at that time and was therefore significantly outdated and flawed. We have since made 2 updates to FlyCyc (Table 3). The first update, in 2023, rectified many of the errors present in the original FlyCyc, resulting in a net decrease in the number of enzymes and enzymatic reactions, but a significant increase in the number of computed metabolic pathways. In turn, these improvements to FlyCyc revealed missing activities within pathways (“pathway holes”), some of which were caused by omission/errors in the GO itself or in *Drosophila* GO annotations. After addressing these issues, a second FlyCyc update was made earlier this year (2025), resulting in a modest, but important, increase in the number of enzymes, reactions, and pathways. FlyCyc is now the most accurate, detailed, and evidence-based metabolic pathway database available for *Drosophila*, thereby enabling the construction of improved *Drosophila* GEMs. FlyCyc is available at https://biocyc.org/DMEL/ and will be described in more detail elsewhere.

**Table 3.** Summary statistics for previous and current versions of FlyCyc.

	Old FlyCyc (2008 August)	Updated FlyCyc (2023 December)	Latest FlyCyc (2025 July)
FlyBase release	FB2008_07	FB2023_06	FB2025_03
Genome annotation version	5.10	6.55	6.64
# Genes	15,097	17,856	17,855
# Enzymes	3,504	2,273	2,277
# Enzymatic reactions	2,561	2,120	2,339
# Pathways	230	316	320

### Summary and future perspectives

Our work directly benefits researchers querying *Drosophila* enzyme annotation data in FlyBase or our partner databases, whether that be on an individual gene/protein level or via bulk analyses such as gene enrichment. The reciprocal interactions in building FlyCyc provided an important cross-validation of the coverage of our enzyme annotations and generated a high-quality metabolic pathway database for the Drosophila community. Enhancements to the FlyBase website, such as exposing the corresponding EC and RHEA annotations and composing enzymatic gene group reports, further aid access to and interrogation of *Drosophila* enzyme data. Moreover, the ontology and annotation improvements stemming from our *Drosophila* review benefits a much wider community since the GO is used to annotate enzyme function across species, and annotations are propagated between taxa. Furthermore, our set of reviewed *Drosophila* enzyme annotations will be useful for training or benchmarking of computational/AI-based functional predictions.

We will continue to monitor the literature and GO annotation pipelines to ensure *Drosophila* enzyme data remain accurate and up-to-date, adding experimental evidence for fly enzyme function wherever possible. We will also periodically update FlyCyc so that it reflects current enzyme annotations.

## Supplementary Material

jkaf294_Supplementary_Data

## Data Availability

All Drosophila enzyme annotation data are freely available at www.flybase.org. GO annotation data are also freely available at several other resources, including www.alliancegenome.org, https://amigo.geneontology.org/amigo, https://www.ebi.ac.uk/QuickGO/. The FlyCyc database is available at https://biocyc.org/DMEL/; access requires a subscription to BioCyc. Supplemental material available at G3 online.

## References

[jkaf294-B1] Abrams EW, Andrew DJ. 2002. Prolyl 4-hydroxylase alpha-related proteins in Drosophila melanogaster: tissue-specific embryonic expression of the 99F8-9 cluster. Mech Dev. 112:165–171. 10.1016/s0925-4773(01)00636-0. 11850189

[jkaf294-B2] Ahn SJ, Marygold SJ. 2021. The UDP-glycosyltransferase family in Drosophila melanogaster: nomenclature update, gene expression and phylogenetic analysis. Front Physiol. 12:648481. 10.3389/fphys.2021.648481. 33815151 PMC8010143

[jkaf294-B3] Alliance of Genome Resources Consortium . 2024. Updates to the alliance of genome resources central infrastructure. Genetics. 227:iyae049. 10.1093/genetics/iyae049. 38552170 PMC11075569

[jkaf294-B4] Attrill H et al 2016. FlyBase: establishing a Gene Group resource for Drosophila melanogaster. Nucleic Acids Res. 44(D1):D786–D792. 10.1093/nar/gkv1046. 26467478 PMC4702782

[jkaf294-B5] Bansal P et al 2022. Rhea, the reaction knowledgebase in 2022. Nucleic Acids Res. 50:D693–D700. 10.1093/nar/gkab1016. 34755880 PMC8728268

[jkaf294-B6] Büchel F et al 2013. Path2Models: large-scale generation of computational models from biochemical pathway maps. BMC Syst Biol. 7:116. 10.1186/1752-0509-7-116. 24180668 PMC4228421

[jkaf294-B7] Carvalho M et al 2010. Survival strategies of a sterol auxotroph. Development. 137:3675–3685. 10.1242/dev.044560. 20940226 PMC2964098

[jkaf294-B8] Cesur MF, Basile A, Patil KR, Çakır T. 2023. A new metabolic model of Drosophila melanogaster and the integrative analysis of Parkinson's disease. Life Sci Alliance. 6:e202201695. 10.26508/lsa.202201695. 37236669 PMC10215973

[jkaf294-B9] Dermauw W, Van Leeuwen T, Feyereisen R. 2020. Diversity and evolution of the P450 family in arthropods. Insect Biochem Mol Biol. 127:103490. 10.1016/j.ibmb.2020.103490. 33169702

[jkaf294-B10] Garapati PV, Zhang J, Rey AJ, Marygold SJ. 2019. Towards comprehensive annotation of Drosophila melanogaster enzymes in FlyBase. Database (Oxford). 2019:bay144. 10.1093/database/bay144. 30689844 PMC6343044

[jkaf294-B11] Gene Ontology Consortium . 2023. The Gene Ontology knowledgebase in 2023. Genetics. 224:iyad031. 10.1093/genetics/iyad031. 36866529 PMC10158837

[jkaf294-B12] Goldberg T, Sreelatha A. 2023. Emerging functions of pseudoenzymes. Biochem J. 480:715–728. 10.1042/BCJ20220373. 37204401 PMC10211241

[jkaf294-B13] Gossmann TI et al 2012. NAD(+) biosynthesis and salvage—a phylogenetic perspective. FEBS J. 279:3355–3363. 10.1111/j.1742-4658.2012.08559.x. 22404877

[jkaf294-B14] Griesemer M, Kimbrel JA, Zhou CE, Navid A, D'haeseleer P. 2018. Combining multiple functional annotation tools increases coverage of metabolic annotation. BMC Genomics. 19:948. 10.1186/s12864-018-5221-9. 30567498 PMC6299973

[jkaf294-B15] Gu C, Kim GB, Kim WJ, Kim HU, Lee SY. 2019. Current status and applications of genome-scale metabolic models. Genome Biol. 20:121. 10.1186/s13059-019-1730-3. 31196170 PMC6567666

[jkaf294-B16] Hu Y et al 2022. Paralog Explorer: a resource for mining information about paralogs in common research organisms. Comput Struct Biotechnol J. 20:6570–6577. 10.1016/j.csbj.2022.11.041. 36467589 PMC9712503

[jkaf294-B17] Kanehisa M, Furumichi M, Sato Y, Matsuura Y, Ishiguro-Watanabe M. 2025. KEGG: biological systems database as a model of the real world. Nucleic Acids Res. 53:D672–D677. 10.1093/nar/gkae909. 39417505 PMC11701520

[jkaf294-B18] Kanzok SM et al 2001. Substitution of the thioredoxin system for glutathione reductase in Drosophila melanogaster. Science. 291:643–646. 10.1126/science.291.5504.643. 11158675

[jkaf294-B19] Karp PD, Latendresse M, Caspi R. 2011. The pathway tools pathway prediction algorithm. Stand Genomic Sci. 5:424–429. 10.4056/sigs.1794338. 22675592 PMC3368424

[jkaf294-B20] Karp PD et al 2019. The BioCyc collection of microbial genomes and metabolic pathways. Brief Bioinform. 20:1085–1093. 10.1093/bib/bbx085. 29447345 PMC6781571

[jkaf294-B21] Lu J, Marygold SJ, Gharib WH, Suter B. 2015. The aminoacyl-tRNA synthetases of Drosophila melanogaster. Fly (Austin). 9:53–61. 10.1080/19336934.2015.1101196. 26761199 PMC4826098

[jkaf294-B22] Manning G, Plowman GD, Hunter T, Sudarsanam S. 2002. Evolution of protein kinase signaling from yeast to man. Trends Biochem Sci. 27:514–520. 10.1016/s0968-0004(02)02179-5. 12368087

[jkaf294-B23] Marygold SJ . 2024. The alpha-ketoacid dehydrogenase complexes of Drosophila melanogaster. MicroPubl Biol. 2024:10.17912/micropub.biology.001209. 10.17912/micropub.biology.001209. PMC11089389 38741935

[jkaf294-B24] Marygold SJ, Alic N, Gilmour DS, Grewal SS. 2020a. In silico identification of Drosophila melanogaster genes encoding RNA polymerase subunits. MicroPubl Biol. 2020:10.17912/micropub.biology.000320. 10.17912/micropub.biology.000320. PMC7704258 33274328

[jkaf294-B25] Marygold SJ, FlyBase Consortium. 2023. Exploring FlyBase data using QuickSearch. Curr Protoc. 3:e731. 10.1002/cpz1.731. 37014762 PMC10088454

[jkaf294-B26] Marygold SJ et al 2020b. The DNA polymerases of Drosophila melanogaster. Fly (Austin). 14:49–61. 10.1080/19336934.2019.1710076. 31933406 PMC7714529

[jkaf294-B27] McDonald AG, Tipton KF. 2023. Enzyme nomenclature and classification: the state of the art. FEBS J. 290:2214–2231. 10.1111/febs.16274. 34773359

[jkaf294-B28] Meyer H et al 2021. Identification and bioinformatic analysis of neprilysin and neprilysin-like metalloendopeptidases in Drosophila melanogaster. MicroPubl Biol. 2021:10.17912/micropub.biology.000410. 10.17912/micropub.biology.000410. PMC8223033 34189422

[jkaf294-B29] Öztürk-Çolak A et al 2024. FlyBase: updates to the Drosophila genes and genomes database. Genetics. 227:iyad211. 10.1093/genetics/iyad211. 38301657 PMC11075543

[jkaf294-B30] Pardue ML, DeBaryshe PG. 2003. Retrotransposons provide an evolutionarily robust non-telomerase mechanism to maintain telomeres. Annu Rev Genet. 37:485–511. 10.1146/annurev.genet.38.072902.093115. 14616071

[jkaf294-B31] Rawlings ND et al 2018. The MEROPS database of proteolytic enzymes, their substrates and inhibitors in 2017 and a comparison with peptidases in the PANTHER database. Nucleic Acids Res. 46:D624–D632. 10.1093/nar/gkx1134. 29145643 PMC5753285

[jkaf294-B32] Reams AB, Roth JR. 2015. Mechanisms of gene duplication and amplification. Cold Spring Harb Perspect Biol. 7:a016592. 10.1101/cshperspect.a016592. 25646380 PMC4315931

[jkaf294-B33] Ribeiro AJM et al 2019. Emerging concepts in pseudoenzyme classification, evolution, and signaling. Sci Signal. 12:eaat9797. 10.1126/scisignal.aat9797. 31409758

[jkaf294-B34] Schnoes AM, Brown SD, Dodevski I, Babbitt PC. 2009. Annotation error in public databases: misannotation of molecular function in enzyme superfamilies. PLoS Comput Biol. 5:e1000605. 10.1371/journal.pcbi.1000605. 20011109 PMC2781113

[jkaf294-B35] Seal RL et al 2023. Genenames.org: the HGNC resources in 2023. Nucleic Acids Res. 51:D1003–D1009. 10.1093/nar/gkac888. 36243972 PMC9825485

[jkaf294-B37] Wang H et al 2021. Genome-scale metabolic network reconstruction of model animals as a platform for translational research. Proc Natl Acad Sci U S A. 118:e2102344118. 10.1073/pnas.2102344118. 34282017 PMC8325244

[jkaf294-B38] Wennerberg K, Rossman KL, Der CJ. 2005. The Ras superfamily at a glance. J Cell Sci. 118:843–846. 10.1242/jcs.01660. 15731001

[jkaf294-B39] Zaru R, Marygold SJ. 2025. *In silico* identification and analysis of paralogs encoding enzymes of carbohydrate metabolism in *Drosophila melanogaster*. MicroPubl Biol. 2025:10.17912/micropub.biology.001425. 10.17912/micropub.biology.001425. PMC11836677 39975507

