# Supplementary material for: Comprehensive annotation of the enzymes of Drosophila melanogaster
Source: G3 (Bethesda). 2025 Dec 8;16(2):jkaf294. doi: 10.1093/g3journal/jkaf294 (PMC12869072; doi:10.1093/g3journal/jkaf294)
Supplement: jkaf294_Supplementary_Data [file jkaf294_supplementary_data.zip › Figure_S1_G3-2025-406285.pdf]

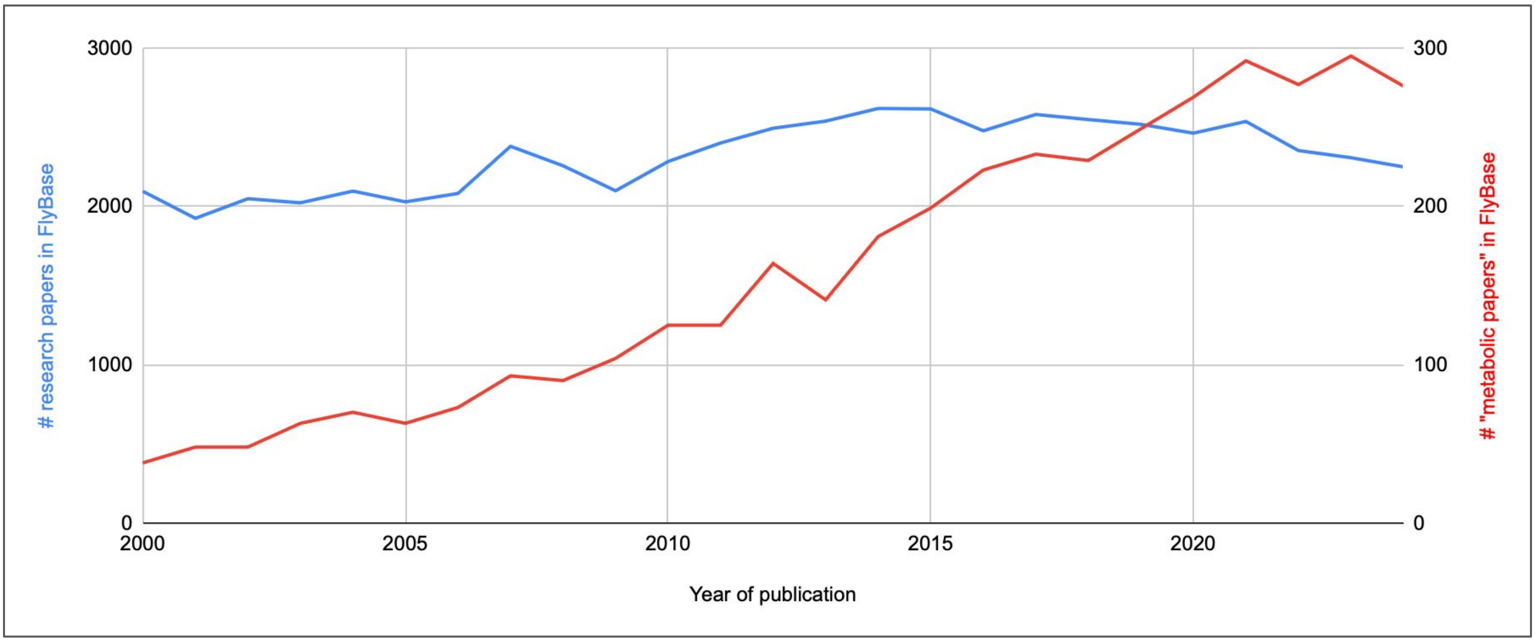

Figure S1. Number of *Drosophila* research papers published per year that included the string 'metaboli*' in their title and/or abstract (red line), compared to the total number of research papers (blue line) during the last 25 years, as recorded in the FlyBase bibliography.
