# Supplementary material for: Comprehensive annotation of the enzymes of Drosophila melanogaster
Source: G3 (Bethesda). 2025 Dec 8;16(2):jkaf294. doi: 10.1093/g3journal/jkaf294 (PMC12869072; doi:10.1093/g3journal/jkaf294)
Supplement: jkaf294_Supplementary_Data [file jkaf294_supplementary_data.zip › Figure_S2_G3-2025-406285.pdf]

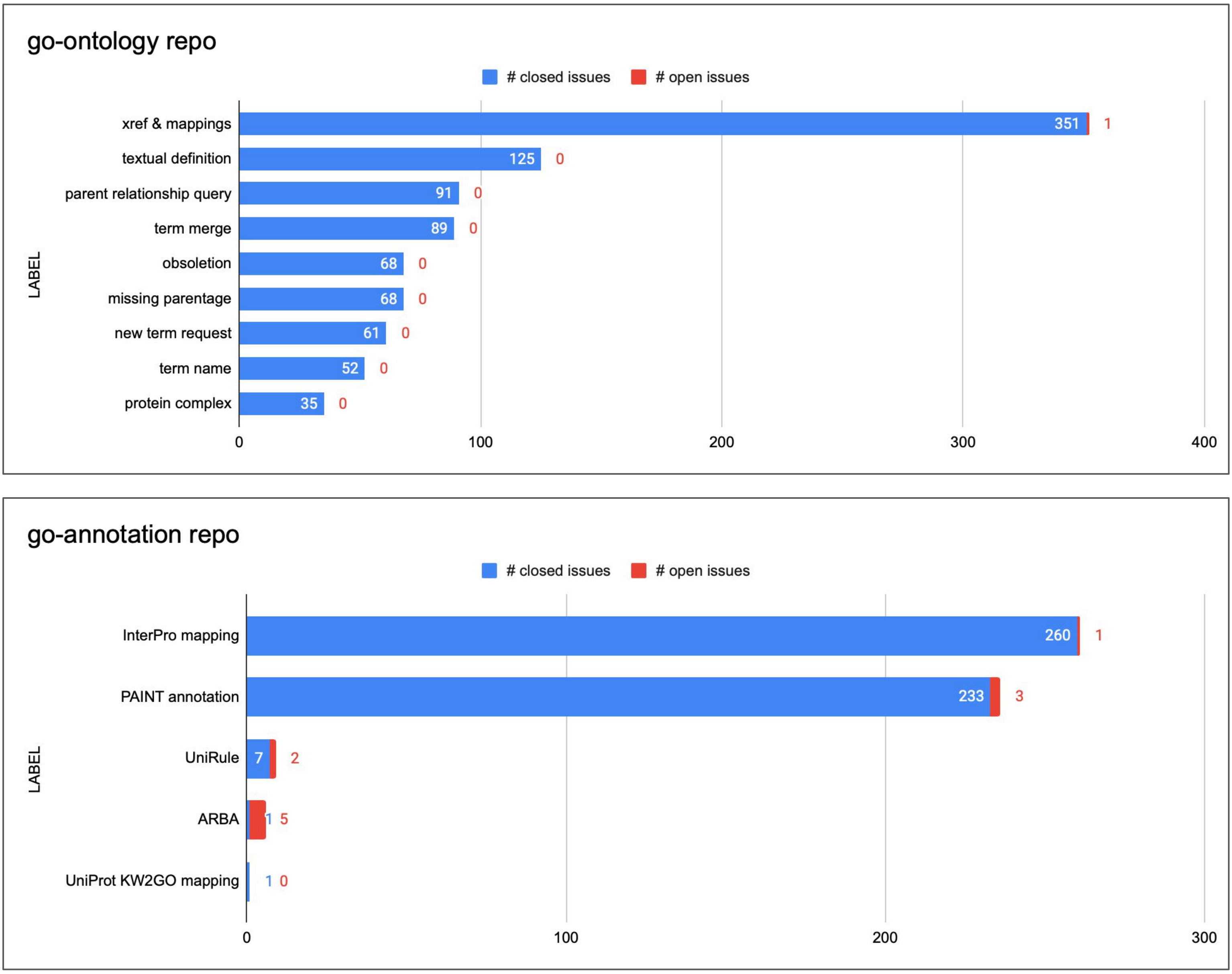

Figure S2. Classification of enzyme-related issues created during this project in the two main Gene Ontology GitHub repositories, go-ontology (for ontology issues) and go-annotation (for annotation issues). Note that a single issue may be tagged with more than one label.
