# Supplementary material for: Comprehensive annotation of the enzymes of Drosophila melanogaster
Source: G3 (Bethesda). 2025 Dec 8;16(2):jkaf294. doi: 10.1093/g3journal/jkaf294 (PMC12869072; doi:10.1093/g3journal/jkaf294)
Supplement: jkaf294_Supplementary_Data [file jkaf294_supplementary_data.zip › Supplemental_Table_legends_G3-2025-406285.docx]

# Supplemental Table Legends

**Table S1.** Catalytic GO annotation comparisons between *Drosophila*, yeast and human. Tab 1: Catalytic GO terms annotated only in *Drosophila*. Tab 2: Catalytic GO terms annotated only in humans & yeast.

**Table S2.** Enzyme-encoding paralogs in Drosophila. Tab 1: All paralog pairs with DIOPT score of 7 or above. Tab 2: Enzyme-encoding paralog pairs with DIOPT score of 7 or above. The testis-specificity index (TSI) is shown, which varies between − 2.52 and 5.2: negative values represent the underrepresentation of transcripts in testis, lower values represent genes expressed in multiple tissues or ubiquitously; values higher than 4 are highly enriched in testis (Vedelek et al. 2018). Tab 3: Enzyme gene group membership of enzyme-encoding genes listed in Tab 2.

**Table S3.** Catalytic complexes in *Drosophila*. Enzyme-encoding genes that have one or more annotations to GO terms that are children of the parent GO term ‘catalytic complex’.

**Table S4.** ‘Orphan’ gene reports in FlyBase representing enzymatic activities at the start of this project (FB2017_05) and their current status (FB2025_04).

**Table S5.** Nomenclature updates for enzyme-encoding genes between the start of this project (FB2017_05) and the current release (FB2025_04).

**References:**

Vedelek V, Bodai L, Grézal G, Kovács B, Boros IM, Laurinyecz B, Sinka R. 2018. Analysis of Drosophila melanogaster testis transcriptome. *BMC Genomics*. 19(1):697. doi:10.1186/s12864-018-5085-z.
